# Supplementary material for: Anionic Phospholipids Stimulate the Proton Pumping Activity of the Plant Plasma Membrane P-Type H+-ATPase
Source: Int J Mol Sci. 2023 Aug 23;24(17):13106. doi: 10.3390/ijms241713106 (PMC10488199; doi:10.3390/ijms241713106)
Supplement: Supplementary file 1 [file ijms-24-13106-s001.zip › ijms-2567807-supplementary.pdf]

International Journal of *Molecular Sciences*

MDPI

Article

Anionic Phospholipids Stimulate the Proton Pumping Activity of the Plant Plasma Membrane P-type H^+-ATPase

Laura C. Paweletz [1], Simon L. Holtbrügge [2], Malina Löb [1], Dario De Vecchis [2], Lars V. Schäfer [2,*], Thomas Günther Pomorski [1,3] and Bo Højen Justesen [1,*]

[1] Department of Molecular Biochemistry, Faculty of Chemistry and Biochemistry, Ruhr University Bochum, 44780 Bochum, Germany; laura.paweletz@ruhr-uni-bochum.de (L.C.P.); malina.loeb@ruhr-uni-bochum.de (M.L.); thomas.guenther-pomorski@ruhr-uni-bochum.de (T.G.P.)

[2] Center for Theoretical Chemistry, Faculty of Chemistry and Biochemistry, Ruhr University Bochum, 44780 Bochum, Germany; simon.holtbruegge@ruhr-uni-bochum.de (S.L.H.); dario.devecchis@ruhr-uni-bochum.de (D.D.V.)

[3] Department of Plant and Environmental Sciences, University of Copenhagen, 1871 Frederiksberg C, Denmark

* Correspondence: lars.schaefer@ruhr-uni-bochum.de (L.V.S.); bo.justesen@ruhr-uni-bochum.de (B.H.J.)

Supplement S

This file includes all supplementary information for the manuscript:
Supplemental Figures S1
Supplemental Tables S1, S2, S3, S4 and S5.

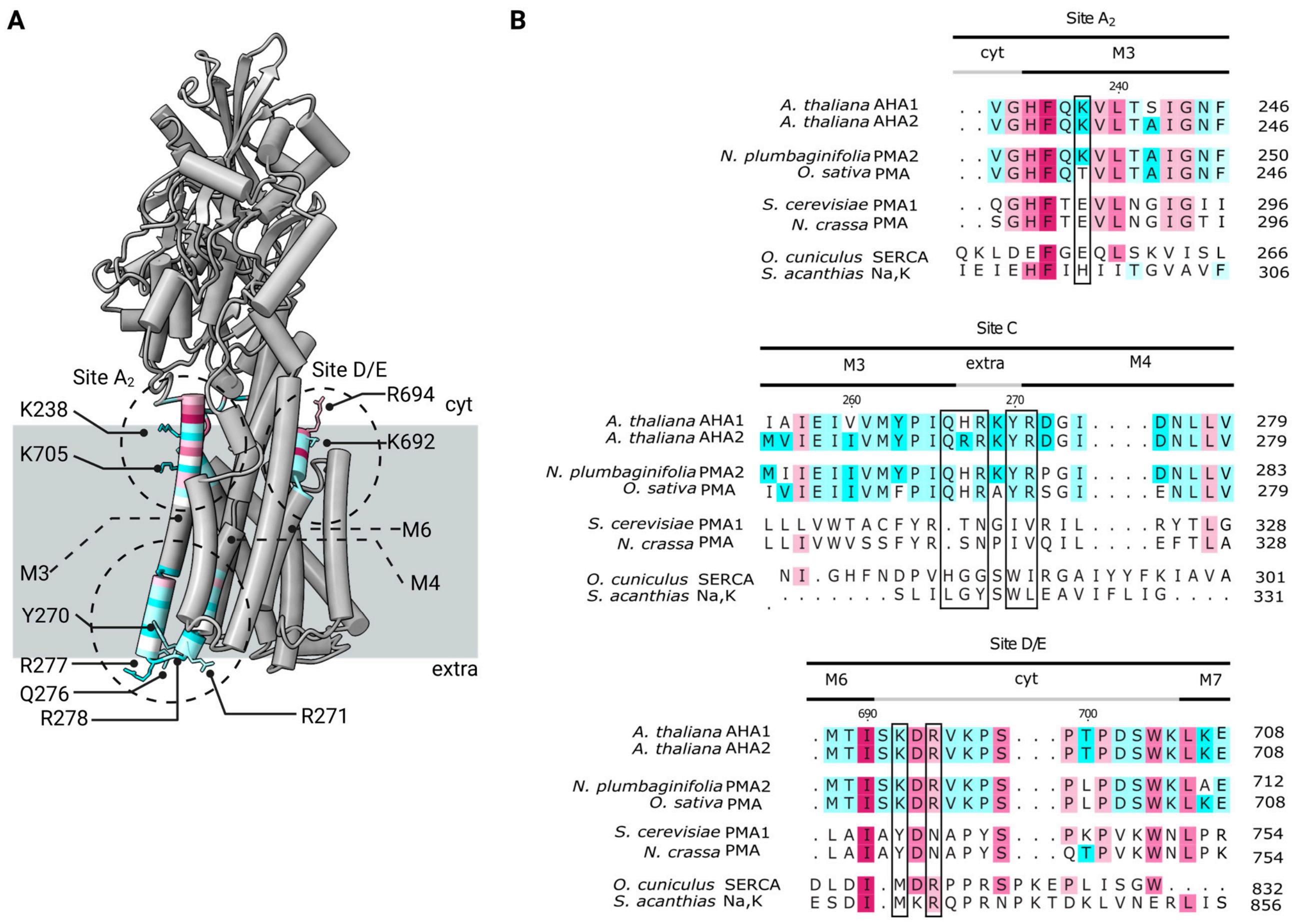

Supplemental Figure S1. Location of predicted phospholipid contact sites B, C, D and E in AHA2. (A) Cartoon representation of the AHA2 membrane domain. Patches of apprx. 15 residues around the assigned contact residues are colored according to their sequence conservation based on AL2CO algorithm implemented into ChimeraX [1]. Preferentially interacting residues identified in the lipid-protein contact analysis are shown in atom representation. The approximate regions of the anionic lipid contacts sites, as identified in the lipid density maps, are marked with black, dashed circles. (B) Sequence alignment showing the conservation of residues based on AL2CO algorithm in selected P-type ATPase proton pumps from plants and yeasts as well as SERCA and Na-K-ATPase at the lipid contact sites A2, C, D and E identified in AHA2. The sequences are obtained from the Uniprot database: *Arabidopsis thaliana* H^+-ATPase isoforms 1 and 2 (P20649 and P19456, respectively), shown in the first block, followed by the plant transporters *Nicotiana plumbaginifolia* H^+-ATPase isoform 2 (Q42932), *Oryza sativa subsp. Japonica* H^+-ATPase (Q7XPY2), and the two fungal transporters (*Saccharomyces cerevisiae* H^+-ATPase isoform 1 (P05030) and *Neurospora crassa* H^+-ATPase (P07038). Lastly, they are compared to the SERCA pump from rabbit *Oryctolagus cuniculus* (P04191) and the Na,K-ATPase from shark *Squalus acanthias* (Q4H132). Residues in black boxes are found to have enriched lipid contacts in the MD simulations.

Supplemental Table S1: Statistical analysis of the data presented in Figure 1. The count column gives the number of reconstitutions resulting in either active or inactive reconstituted proton pump (Plateau after magnesium addition below 10% of the CCCP plateau, see Material and Methods). The mean of indicated measurements (active counts) is displayed together with standard deviation. Data was undertaken pairwise t-test and obtained p-values were group – p > 0.05: not significant (ns), p < 0.05: significant (*), p < 0.01: very significant (**), p < 0.001: highly significant (***)

	Count (active)	Count (inactive)	mean	std	PC	PE	PA	PG	PS	Mix	group
PC	12	2	15.7	7.7	-	ns	ns	*	***	**	b
PE	4	2	11.9	5.5		-	ns	ns	***	**	b
PA	3	1	31.8	5.4			-	ns	***	ns	bc
PG	8	1	34.9	9.3				-	***	ns	bc
PS	9	-	82.5	22.5					-	**	a
Mix	5	-	49.1	14.4						-	c

Supplemental Table S2: S**tatistical analysis of the data presented in Figure 2A (PA titration).** The count column gives the number of reconstitutions resulting in either active or inactive reconstituted proton pump (Plateau after magnesium addition below 10% of the CCCP plateau, see Material and Methods). The mean of indicated measurements (active counts) is displayed together with standard deviation. Data was undertaken pairwise t-test and obtained p-values were group – p > 0.05: not significant (ns), p < 0.05: significant (*), p < 0.01: very significant (**), p < 0.001: highly significant (***)

	Count (active)	Count (inactive)	mean	std	0 mol%	10 mol%	20 mol%	30 mol%	group
0 mol%	12	2	15.7	7.7	-	ns	**	ns	a
10 mol%	4	-	27.6	15.0		-	ns	ns	ab
20 mol%	2	-	50.5	16.6			-	ns	b
30 mol%	3	1	31.8	5.4				-	ab

Supplemental Table S3: Statistical analysis of the data presented in Figure 2B (PG titration). The count column gives the number of reconstitutions resulting in either active or inactive reconstituted proton pump (Plateau after magnesium addition below 10% of the CCCP plateau, see Material and Methods). The mean of indicated measurements (active counts) is displayed together with standard deviation. Data was undertaken pairwise t-test and obtained p-values were group – $p > 0.05$: not significant (ns), $p < 0.05$: significant (*), $p < 0.01$: very significant (**), $p < 0.001$: highly significant (***)

	Count (active)	Count (inactive)	mean	std	0 mol%	10 mol%	20 mol%	30 mol%	group
0 mol%	12	2	15.7	7.7	-	ns	**	**	a
10 mol%	3	-	22.5	14.7		-	ns	ns	ab
20 mol%	3	-	42.5	13.6			-	ns	b
30 mol%	8	1	34.9	9.3				-	b

Supplemental Table S4: Statistical analysis of the data presented in Figure 2C (PS titration). The count column gives the number of reconstitutions resulting in either active or inactive reconstituted proton pump (Plateau after magnesium addition below 10% of the CCCP plateau, see Material and Methods). The mean of indicated measurements (active counts) is displayed together with standard deviation. Data was undertaken pairwise t-test and obtained p-values were group – $p > 0.05$: not significant (ns), $p < 0.05$: significant (*), $p < 0.01$: very significant (**), $p < 0.001$: highly significant (***)

	Count (active)	Count (inactive)	mean	std	0 mol%	10 mol%	20 mol%	30 mol%	group
0 mol%	12	2	15.7	7.7	-	ns	***	***	b
10 mol%	4	1	31.6	13.5		-	*	***	b
20 mol%	3	-	68.2	11.3			-	ns	a
30 mol%	9	-	82.5	22.5				-	a

Supplemental Table S5. Percentage of time protein residues are in contact with lipid headgroups. For PS headgroups, results are given for all three independent repeats and their average. For neutral headgroups, only the average is given. The tables are sorted by average PS contacts, including only residues with ≥5% PS contacts on average. Residues which show no specificity towards PS lipids are greyed out, with a ratio of average neutral:PS contacts of 80:20 or less; 90:10 is the lipid mixture.

PC:PS (90:10)

Name	ID	PS				neutral
		run 1	run 2	run 3	Av.	Av.
Arg	271	28	26	29	27	71
Tyr	270	26	26	27	26	42
Lys	707	21	25	32	26	74
Arg	267	21	26	27	24	60
Lys	60	30	17	21	23	77
Arg	268	26	19	22	22	58
Lys	57	28	15	19	21	39
Phe	61	25	14	21	20	60
Lys	692	19	20	18	19	79
Lys	705	14	21	22	19	38
Lys	238	21	12	23	19	34
Trp	667	19	19	19	19	66
Lys	733	17	29	10	19	62
Trp	66	22	13	19	18	66
Arg	842	11	20	22	18	61
Leu	706	19	12	20	17	28
Arg	694	15	17	19	17	63
Gln	266	17	15	17	17	79
Asn	67	12	12	15	13	76
Thr	241	12	10	18	13	57
Asn	245	13	9	17	13	64
Thr	734	12	21	5	13	76
Tyr	804	12	10	16	13	75
Gly	816	9	12	15	12	46
Trp	817	9	12	14	12	69
Ile	666	12	14	10	12	40
Val	743	8	14	12	12	73
Lys	269	14	9	11	11	18
Ala	785	10	11	13	11	47
Arg	89	9	9	16	11	88
Asn	85	11	8	14	11	76
Thr	689	10	10	12	11	45
Glu	708	5	13	13	11	33
Ser	691	11	11	10	11	63
Leu	786	10	11	10	10	35

PC:PE:PS (45:45:10)

Name	ID	PS				neutral
		run 1	run 2	run 3	Av.	Av.
Lys	707	29	28	27	28	71
Arg	842	21	22	26	23	58
Lys	705	22	23	20	22	41
Lys	238	20	23	18	20	43
Arg	271	26	14	14	18	80
Arg	694	12	18	21	17	63
Lys	733	19	12	20	17	60
Leu	706	18	19	13	17	29
Lys	60	21	17	9	16	75
Arg	268	21	9	18	16	55
Arg	267	22	12	13	16	75
Trp	667	15	11	18	14	71
Gly	816	13	11	16	14	46
Phe	61	17	16	6	13	67
Thr	241	12	11	16	13	62
Thr	734	14	10	13	13	77
Gln	266	15	9	14	13	83
Lys	57	14	21	3	13	35
Tyr	270	23	7	7	12	52
Pro	783	11	9	14	12	63
Trp	66	15	13	6	12	68
Trp	817	11	9	13	11	70
Glu	708	10	10	13	11	29
Asn	245	10	11	13	11	58
Asn	85	11	12	11	11	77
Arg	89	8	14	10	11	88
Arg	782	13	8	11	11	52
Trp	729	12	7	12	10	42
Ala	785	6	12	12	10	48
Arg	744	9	7	13	10	25
Val	743	11	7	12	10	75
Val	803	10	8	11	10	39
Tyr	804	11	8	10	10	78
Ile	666	10	6	12	9	41
Asn	67	10	9	9	9	78

Phe	710	11	8	11	10	27
Trp	729	9	15	6	10	39
Asn	806	12	4	13	10	87
Phe	64	14	6	8	9	23
Gly	784	8	9	12	9	42
Pro	783	8	10	10	9	69
Arg	744	7	11	10	9	26
Met	688	9	10	9	9	58
Leu	59	11	9	8	9	42
Asn	115	10	10	6	9	69
Ile	265	8	9	9	9	40
Val	803	7	8	11	9	40
Ala	242	8	6	11	9	19
Phe	111	12	8	5	8	45
Gly	818	6	11	8	8	40
Asn	116	10	6	9	8	69
Tyr	843	6	9	8	7	83
Phe	736	7	14	1	7	72
Phe	669	7	11	4	7	18
Thr	740	7	11	3	7	73
Trp	71	6	6	9	7	46
Phe	741	6	8	6	7	74
Ser	776	6	9	5	6	73
Gly	86	6	6	7	6	20
Asp	735	5	12	1	6	70
Phe	839	5	9	5	6	61
Met	65	8	3	7	6	17
Gly	742	4	8	5	6	61
Ala	805	7	3	8	6	33
Arg	813	6	3	8	6	15
Glu	781	6	8	4	6	82
Arg	782	5	7	5	6	44
Asp	693	5	6	5	6	13
Asp	739	5	9	2	5	86
His	235	6	4	6	5	4

Gly	784	6	10	11	9	44
Lys	692	11	8	7	9	89
Phe	710	10	9	7	8	25
Ala	242	9	8	7	8	20
Glu	781	8	7	9	8	81
Leu	59	10	7	7	8	51
Asn	115	9	9	5	8	67
Asn	116	10	6	6	7	70
Tyr	843	6	7	9	7	83
Ile	265	10	5	6	7	50
Ser	776	5	8	8	7	72
Phe	839	5	7	8	7	60
Gly	818	6	5	9	7	36
His	235	7	8	4	6	4
Phe	741	5	6	6	6	77
Gly	742	5	6	6	6	67
Val	780	6	5	5	6	81
Gly	814	5	5	7	5	11
Asn	806	7	4	5	5	91
Phe	669	6	2	8	5	16
Trp	71	7	4	6	5	45
Leu	786	5	6	5	5	44
Ile	815	5	5	6	5	11
Ile	844	5	5	6	5	78
Gly	86	7	4	5	5	26
Trp	93	3	7	5	5	42

References

1. Pei, J.; Grishin, N.V. AL2CO: Calculation of Positional Conservation in a Protein Sequence Alignment. *Bioinformatics* **2001**, *17*, 700–712, doi:10.1093/bioinformatics/17.8.700.
